# Regulation of stem cell self-renewal and differentiation by Wnt and Notch are conserved throughout the adenoma-carcinoma sequence in the colon

**DOI:** 10.1186/1476-4598-12-126

**Published:** 2013-10-21

**Authors:** Pramudita Ramadhina Prasetyanti, Cheryl Doreen Zimberlin, Michael Bots, Louis Vermeulen, Felipe De Sousa E Melo, Jan Paul Medema

**Affiliations:** 1 Laboratory of Experimental Oncology and Radiobiology, Center for Experimental Molecular Medicine, Academic Medical Center, Room G2-131, Meibergdreef 9, 1105AZ Amsterdam, The Netherlands; 2 Current affiliation: Cancer Research UK, Cambridge Research Institute, University of Cambridge, Li Ka Shing Centre, Robinson Way, Cambridge, UK

**Keywords:** Colorectal cancer, Wnt, Notch, Stem cells, Adenoma, Morphogenic pathways, Organoid cultures

## Abstract

**Background:**

Colon cancer stem cells are shown to be the self-renewing cells within a tumor that give rise to all lineages of more differentiated tumor cells. In this respect they are remarkably similar to their non-malignant counterparts that orchestrate the intestinal lining. This suggests that, despite the numerous genetic aberrations and morphological changes that have occurred during cancer initiation and progression, a remnant homeostatic regulation persists.

**Findings:**

Using a number of human and mouse intestinal-derived organoid cultures from normal, adenoma and cancerous tissues, we show here that Notch signals coordinate self-renewal and lineage determination not only in normal, but also at the adenoma and carcinoma stage in both mice and humans. Moreover, the Wnt pathway, which carries activating mutations in virtually all colon cancers, is not as previously predicted constitutively active in adenomas and carcinomas, but still displays a heterogeneous activity pattern that determined stemness in all stages of disease.

**Conclusion:**

These data for the first time provide a comprehensive overview of Wnt and Notch-mediated signaling in the different stages of the adenoma-carcinoma sequence and demonstrates that these morphogenic pathways, despite mutations, remain crucial determinants of both architecture and hierarchy in normal and malignant intestinal tissue.

## Introduction

Normal gut homeostasis is ensured by intestinal stem cells (ISCs), which rely on integration of various morphogenic pathways including bone morphogenetic protein, Hedgehog, Notch and Wnt signaling cascades [1]. Colorectal cancer (CRC) is a multistep process in which genetic hits induce transition from normal mucosa via adenomatous lesions to an invasive carcinoma [2]. Adenoma initiation is suggested to occur via deregulation of these morphogenic pathways. First and foremost, mutations leading to activation of the Wnt cascade are seen in all CRC patients [1,3]. However, these activating mutations never result in a complete activation, but rather in a finely-tuned Wnt activity level [4]. Furthermore, cell-to-cell variation in pathways activity is frequently observed. In agreement with these concepts are our recent observations that have pinpointed Wnt pathway regulation as the basis of cancer stemness in CRC [5]. Functionally marked by high Wnt pathway activity, colon cancer stem cells (CSCs) have self-renewal capacity and the potential to differentiate into all cell lineages present in cancerous tissue [6]. These similarities between ISCs and CSCs may therefore point to the intriguing possibility that the response to morphogenic signals is not lost during tumorigenesis. Despite the wealth of data regarding the role of morphogenic pathways in controlling cell fate and proliferation in intestinal tissue, to our surprise few studies have attempted to comprehensively assess their role throughout the stages of neoplastic progression. For example, whereas Notch inhibition leads to goblet cells accumulation in adenomas [7], little is known on the role of Wnt signaling in this stage.

Using a number of human and mouse intestinal-derived organoid cultures from normal, adenoma and cancerous tissues [8,9], we provide a comprehensive overview on the role of both Wnt and Notch morphogenic pathways in the different stages of CRC development. We find that Notch inhibition decreases stemness and enhances goblet-like differentiation in all stages of disease. Conversely, Wnt activity is associated with stemness in normal, adenoma and carcinoma tissue. Our results point to the conclusion that colon carcinogenesis, at early and late stages consistently retain numerous characteristics of the normal intestine.

## Findings

**Figure 1:**
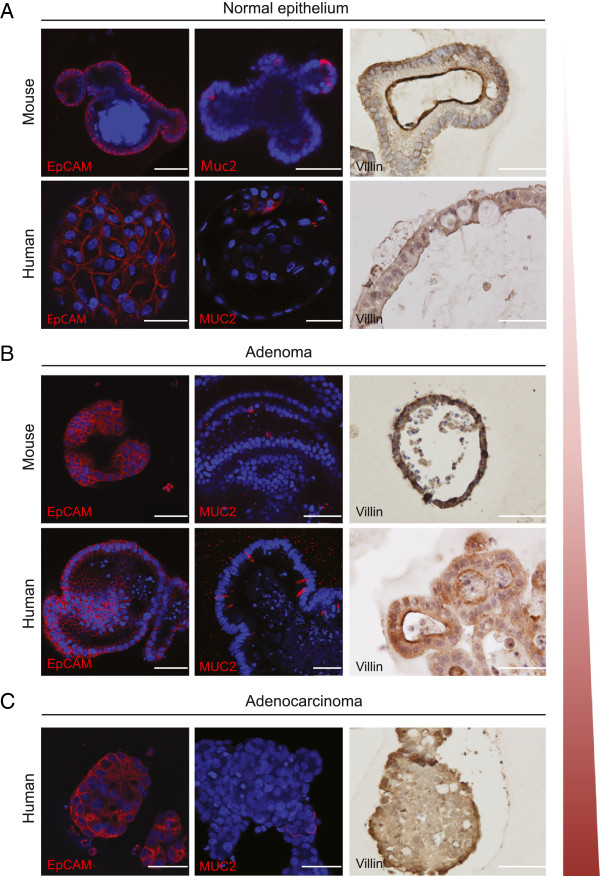
**Multi-lineage potential of intestinal cultures from multiple stages of CRC development.** A representative overview of confocal and immunohistochemistry images displaying epithelial cells (EpCAM), goblet cells (MUC2) and enterocytes (Villin) in organoid cultures of **(A)** healthy epithelial derived from small intestine (mouse) and colon (human), **(B)** adenoma **(C)** adenocarcinoma (human). Scale bar: 50 μm.

Normal intestinal epithelial cultures rapidly self-organized into typical single layered organoid structures composed of epithelial cells (EpCAM^+^) surrounding a central lumen (Figure 1). As previously shown, organoids contained the various differentiated gut cell types, including neuroendocrine cells (Chromogranin A, (CHGA)) (Additional file 1: Figure S1A), goblet cells (MUC2) and enterocytes (Villin) (Figure 1A) [8,9]. Adenoma cultures contained scattered goblet-like cells judged by MUC2 staining (Figure 1B), but appeared to lack CHGA^+^ cells (Additional file 1: Figure S1A) consistent with previous observations [10] and stainings on the corresponding primary tumor material (Additional file 1: Figure S1B). Intriguingly, when analyzing CRC spheroid cultures, derived from a single cell clone and reportedly enriched in CSCs [11], we detected the presence of various lineages (Figure 1C) including the CHGA^+^ neuroendocrine cell lineage. This demonstrates that differentiation towards the distinct epithelial lineages occurs even in late stage cancers and, whenever present, this process might be similarly orchestrated at all stages of the disease.

**Figure 2:**
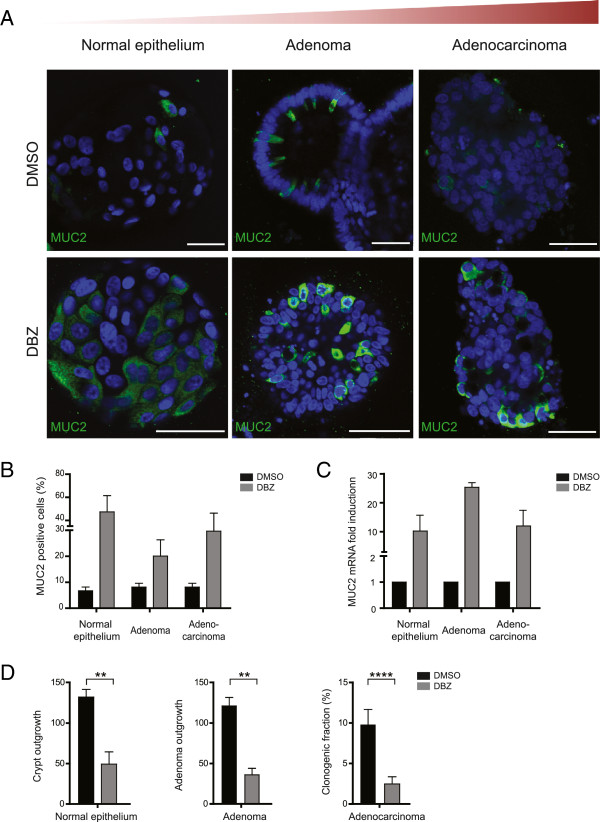
**The Notch pathway is analogously orchestrated throughout CRC progression. (A)** Representative confocal microscopy images of MUC2 (green) in human cultures treated with 10 μM dibenzazepine (DBZ) show differentiation into the goblet cell-lineage as depicted by increased MUC2 expression **(B)** The number of goblet cells was quantified by counting the number of MUC2^+^ cells from 10 random fields at 63× magnification. Values shown are the percentages of MUC2^+^ cells relative to the total number of cells. **(C)** The increased MUC2 expression was further measured with qRT-PCR. *MUC2* mRNA levels were normalized to *GAPDH* mRNA and expressed as fold induction compared with DMSO control. The graph represents the mean ± SD of three different experiments. **(D)** DBZ treatment results in reduced clonogenicity. The results depicted are representative from three independent experiments from human healthy colon organoid (N=2), adenoma (N=3) and adenocarcinoma (N=3). Scale bar: 50 μm. * p<0.05, ** p<0.01, ***p<0.001, ****p<0.0001 (t-test).

To address this, we first assessed the effect of pharmacological inhibition of the Notch pathway in the distinct cultures. As previously reported, dibenzazepine (DBZ) mediated Notch inhibition resulted in goblet cell differentiation in cultured mouse intestinal epithelium and adenoma (Additional file 2: Figure S2). Importantly, also treatment of human epithelial organoid cultures derived either from normal, adenoma or adenocarcinoma tissues with DBZ, caused a clear increase in goblet cells (Figure 2A-B) and a significant induction of *MUC2* mRNA level (Figure 2C). This goblet cell-like differentiation appeared to be observed only in cultures already expressing low amounts of MUC2 (Additional file 3: Figure S3). More importantly, the clonogenic potential of all cultures was clearly decreased upon DBZ treatment, which is consistent with the loss of (cancer) stem cells (Figure 2D and Additional file 2: Figure S2), implying that Notch pathway-dependent self-renewal and cell fate decision are maintained at all stages of tumor development.

**Figure 3:**
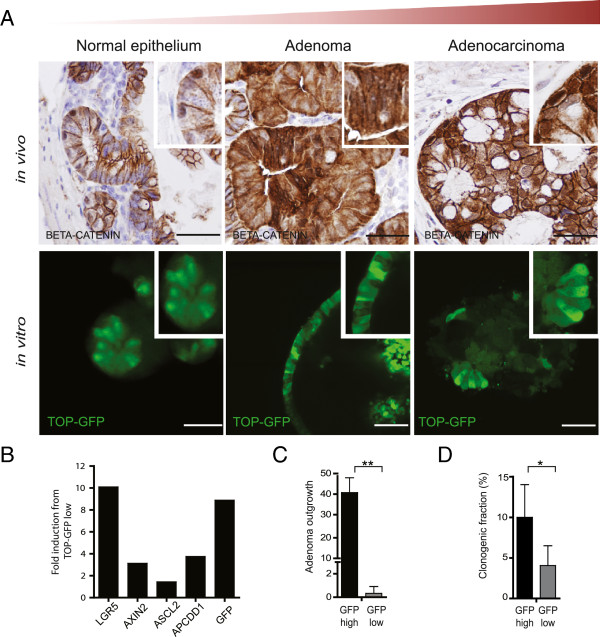
**Functional Wnt activity is maintained throughout CRC development. (A)** Immunohistochemistry staining from mouse intestinal epithelial, mouse adenoma, and human adenocarcinoma sections show a heterogeneous β-catenin intracellular distribution indicating Wnt signaling hierarchy *in vivo* (upper panel). Images are taken at 20× magnification. Lentiviral transduction of TOP-GFP reporter in the cultures derived from the corresponding tissues shows the heterogeneity of Wnt activity *in vitro* (lower panel). The confocal microscopy images are taken at 63× magnification. **(B)** Representative graph of qRT-PCR analysis from TOP-GFP^high^ and TOP-GFP^low^ fractions of mouse adenoma cultures. The mRNA values of several Wnt target genes and stem cell markers were first normalized with GAPDH mRNA and expressed as fold induction compared to TOP-GFP^low^. **(C** and **D)** Colony-forming efficiency in the sorted TOP-GFP cells showing that TOP-GFP^high^ cells exhibit higher clonogenic potential in both mouse **(C)** and human **(D)** adenoma cultures. Data shown represents mean ± SD from three independent experiments from mouse adenoma cultures (N=2) and human adenoma cultures (N=2). Scale bar, 50 μm. * p<0.05, ** p<0.01, ***p<0.0001 (t-test).

Secondly, we carefully assessed the Wnt pathway activity in all derived cultures using the TCF/LEF reporter construct (TOP-GFP) [5]. As mentioned above, deregulation of the Wnt pathway is frequently observed in CRC development and we have shown that high Wnt activity can be used as a functional CSCs marker in adenocarcinomas [5]. In addition, recent reports have highlighted the heterogeneous expression of the Wnt target and stem cell marker LGR5 in both adenomas and carcinomas [12,13]. As expected, a hierarchy in Wnt pathway activity in healthy epithelial and adenocarcinoma organoid cultures could be detected by distinct GFP patterning (Figure 3A). Intriguingly, adenoma cultures also display heterogeneous Wnt activity, with TOP-GFP^high^ adenoma cells displaying high levels of stem cell marker genes. More importantly, TOP-GFP^high^ adenoma cells represented the clonogenic fraction. This indicates that the functional hierarchy of the Wnt pathway, which is known for normal epithelium and adenocarcinoma CSCs, also identifies stemness in adenomas (Figure 3C-D).

## Discussion

Previous studies have highlighted the role of Wnt and/or Notch in distinct stages of colon cancer. For instance, Notch inhibition has been shown to induce goblet differentiation in mouse adenomas and block stemness in colon CSCs [7,14]. Similarly, we have previously shown that Wnt activity determines stemness in adenocarcinoma-derived colon CSCs. This present study, however, for the first time provides a comprehensive overview of these pathways at all stages and extends these surprising findings by showing that Wnt pathway heterogeneity persists at all stages despite evident mutations in the Wnt pathway and that Notch signals maintain a balance between self-renewal and cell lineage decisions. Currently, the molecular mechanisms underlying such heterogeneity remain unclear. Nevertheless, the organization appears to be purely intrinsic to epithelial cells as the mesenchymal niche is not present *in vitro.* Differential Wnt activity in the normal epithelial organoid cultures was suggested to exist due to the presence of Wnt/Notch ligand producing paneth cells [15]. Likewise, tuning of Wnt signaling in adenoma or adenocarcinoma might be achieved by a similar mechanism. In this respect, it is interesting to note that paneth-like cells have been reported in mouse adenomas and reside in close proximity to the LGR5^+^ adenoma stem cells [13]. Furthermore, microenvironment derived signals such as HGF and Jagged-1 [5,16] could also be responsible for this Wnt hierarchy.

## Conclusion

**Figure 4:**
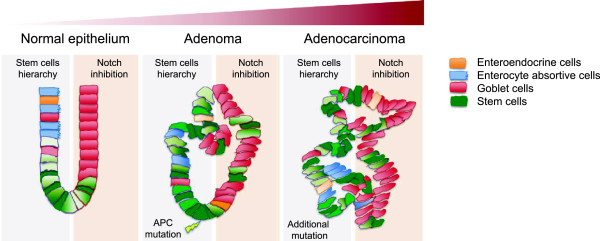
**The signaling architecture defining self-renewal and lineage specification is maintained in colon cancer progression.** Although genetic hits accumulate during progression (e.g. depicted as APC mutation in adenoma), signals defining self-renewal and lineage specification are orchestrated similarly between normal and cancerous tissues. On the left part of each progression step, stemness potential is defined by a gradient of Wnt signals (green, high (dark green) in normal stem cells and cancer stem cells and low (light green) in differentiated (tumor) cells). The right part of each progression step depicts the response to a specific morphogenic cue; in this case inhibition of Notch signaling promotes differentiation towards goblet cells (pink).

Our data on a unique panel of cultures, representing normal, pre-malignant neoplastic lesions as well as adenocarcinoma tissue from both mouse and human origin, shows that at all stages of disease, the tissue is organized in a highly similar hierarchical fashion and present with cell types encompassing multiple differentiation lineages. Here we find that the Wnt and Notch pathways are not only crucial for the orchestration of this hierarchy but their function in regulating self-renewal and cell fate is also conserved during cancer development. Furthermore, our data provide evidence that the robust mechanism orchestrating hierarchy in normal intestinal epithelium is strongly wired in the epithelial cells and its regulation is sustained throughout carcinogenesis (Figure 4). The Wnt and Notch pathways, even though subjected to mutation or aberrant regulation, maintain their central position in the regulation of this hierarchy. These parallels between normal and neoplastic tissue notably makes targeted therapies towards these pathways challenging, as it will predictably impact normal hierarchy. Understanding similarities, but above all the differences in molecular mechanisms will help in highlighting crucial differences that can be exploited for therapeutic design.

## Materials and methods

### Murine tissue culture

The Animal Experimental Committee (DEC) of the Academic Medical Center (AMC) approved all animal experiments. Normal crypt (C57B7/6) and adenoma cultures (Lgr5-EGFP-IRES-creER^T2^/APC^flox/flox^) mice cultures were generated and maintained as previously described by [8] and [17] respectively.

### Human tissue culture

Human tissues were obtained in accordance with the legislation in the Netherlands and approved by the medical ethical committee in the AMC. Human crypt, adenoma and carcinoma cultures were generated and maintained as previously described [9,18].

### Dibenzazepine treatment and clonogenicity assays

For clonogenic assays 150 crypts and 5000 adenoma calls were plated per well in triplicate and outgrowth was measured after 3 days and 7 days respectively of 10 μM dibenzazepine (DBZ) (Merck) treatment. For statistical analysis two-tailed t-tests were applied. Clonogenicity for adenocarcinomas was determined in a limiting dilution fashion at 1, 2, 4, 8, 16, 32, 64 and 128 cells per well in a ultra-low adherent 96 well plate (Corning) after 10 μM DBZ treatment. Results were statistically evaluated using the R software package (http://bioinf.wehi.edu.au/software/elda/index.html).

### Lentiviral transduction

Organoid transductions, as in [19], were performed using TCF/LEF reporter driving expression of GFP (TOP-GFP) was a gift from Laurie Ailles. For clonogenicity assays, 5000 cells were sorted (FACSAria, BD) TOP-GFPhigh and TOP-GFPlow.

### RT-qPCR analysis

Total RNA was extracted using the RNeasy kit (Qiagen) and cDNA was synthesized using SuperScript® III reverse transcriptase (Life Technologies) with random primers (Invitrogen) using 2xSYBR Green Master Mix (Roche) for RT-qPCR measurement. For primers see Additional file 4.

### Immunohistochemistry and immunofluorescence

Immunohistochemistry stainings were analyzed in formalin-fixed, paraffin-embedded tissues and analyzed using standard techniques. 8-chamber culture slides (BD) were used for whole mount immunofluorescent stainings. Images were taken on a Leica TCS-SP2. For antibodies see Additional file 4.

## Abbreviations

ISC: Intestinal stem cells; CSC: Cancer stem cell; CHGA: Chromogranin A; CRC: Colorectal cancer; DBZ: Dibenzazepine; EpCAM: Epithelial cell adhesion molecule; ISC: Intestinal stem cells; MUC2: Mucin2.

## Competing interests

The authors declare that they have no competing interests.

## Authors’ contributions

PRP and CDZ: provision of study material, study design, collection and assembly of data, data analysis and interpretation, manuscript writing. MB and LV: Conception and design, study supervision. FDSM: Conception and design, data analysis and interpretation, manuscript writing. JPM: Conception and design, and supervision, data analysis and interpretation, manuscript writing, final approval of manuscript. All authors read and approved the final manuscript.

## Supplementary Material

Additional file 1: Figure S1 Marker expression of various cell lineages during different stages of CRC development. Click here for file

Additional file 2: Figure S2 Induction of goblet cells in mouse organoid cultures upon DBZ treatment in normal mouse epithelial cells and adenoma organoid cultures. Click here for file

Additional file 3: Figure S3 Induction of goblet cells following DBZ treatment correlates with basal MUC2 expression. Click here for file

Additional file 4: Table S1 RT-qPCR primers. **Table S2**: Antibody list. Click here for file
