# Supplementary material for: Regulation of stem cell self-renewal and differentiation by Wnt and Notch are conserved throughout the adenoma-carcinoma sequence in the colon
Source: Mol Cancer. 2013 Oct 21;12:126. doi: 10.1186/1476-4598-12-126 (PMC4016508; doi:10.1186/1476-4598-12-126)
Supplement: Additional file 1: Figure S1 — Marker expression of various cell lineages during different stages of CRC development. [file 1476-4598-12-126-S1.doc]

**Supplementary Tables**

**Table 1: RT-qPCR primers**

| **Name** | **Forward Primer (‘5 🡪3’)** | **Reverse Primer (‘5 🡪3’)** |
| --- | --- | --- |
| Human GAPDH | CTCATGACCACAGTCCATGC | CACATTGGGGGTAGGAACAC |
| Human MUC2 | CGAAACCACGGCCACAACGT | GACCACGGCCCCGTTAAGCA |
| Murine GAPDH | CTCATGACCACAGTCCATGC | CACATTGGGGGTAGGAACAC |
| Murine Muc2 | CTGACCAAGAGCGAACACAA | CATGACTGGAAGCAACTGGA |
| Murine Lgr5 | TTCGTAGGCAACCCTTCTCT | TCCTGTCAAGTGAGGAAATTCA |
| Murine Axin2 | CCATGACGGACAGTAGCGTA | CTGCGATGCATCTCTCTCTG |
| Murine APCDD1 | GACGCTACCCAGAGGGTGT | TATGGGAGGGTGGTGTTCAT |
| GFP | CGACCACTACCAGCAGAACACCCC | TCGTCCATGCCGAGAGTGATCC |

**Table 2: Antibody list**

| **Primary antibodies** | | |
| --- | --- | --- |
| **Name (Clone)** | **Company** | **Human/Murine Material** |
| EPCAM-FITC | Dako | Human |
| CHGA | Dako | Human |
| Villin (c-19) | Santa Cruz | Human |
| Mucin2 (ccp58) | Santa Cruz | Human |
| EpCAM-PE | Miltenyi | Murine |
| CHGA | Santa Cruz | Murine |
| Villin (c-19) | Santa Cruz | Murine |
| Mucin2 (H300) | Santa Cruz | Murine |
| **Secondary immunofluorescence antibodies** | | |
| **Name** | **Company** | **Dye Conjugate** |
| Goat anti-rabbit (IgG) | Invitrogen | AlexaFluor ® 488 |
| Rabbit anti-mouse (IgG) | Invitrogen | AlexaFluor ® 488 |
