# Supplementary figures and images for: Regulation of stem cell self-renewal and differentiation by Wnt and Notch are conserved throughout the adenoma-carcinoma sequence in the colon
Source: Mol Cancer. 2013 Oct 21;12:126. doi: 10.1186/1476-4598-12-126 (PMC4016508; doi:10.1186/1476-4598-12-126)

**Supplementary figure 1.** Multipotential differentiation from various CRC stages

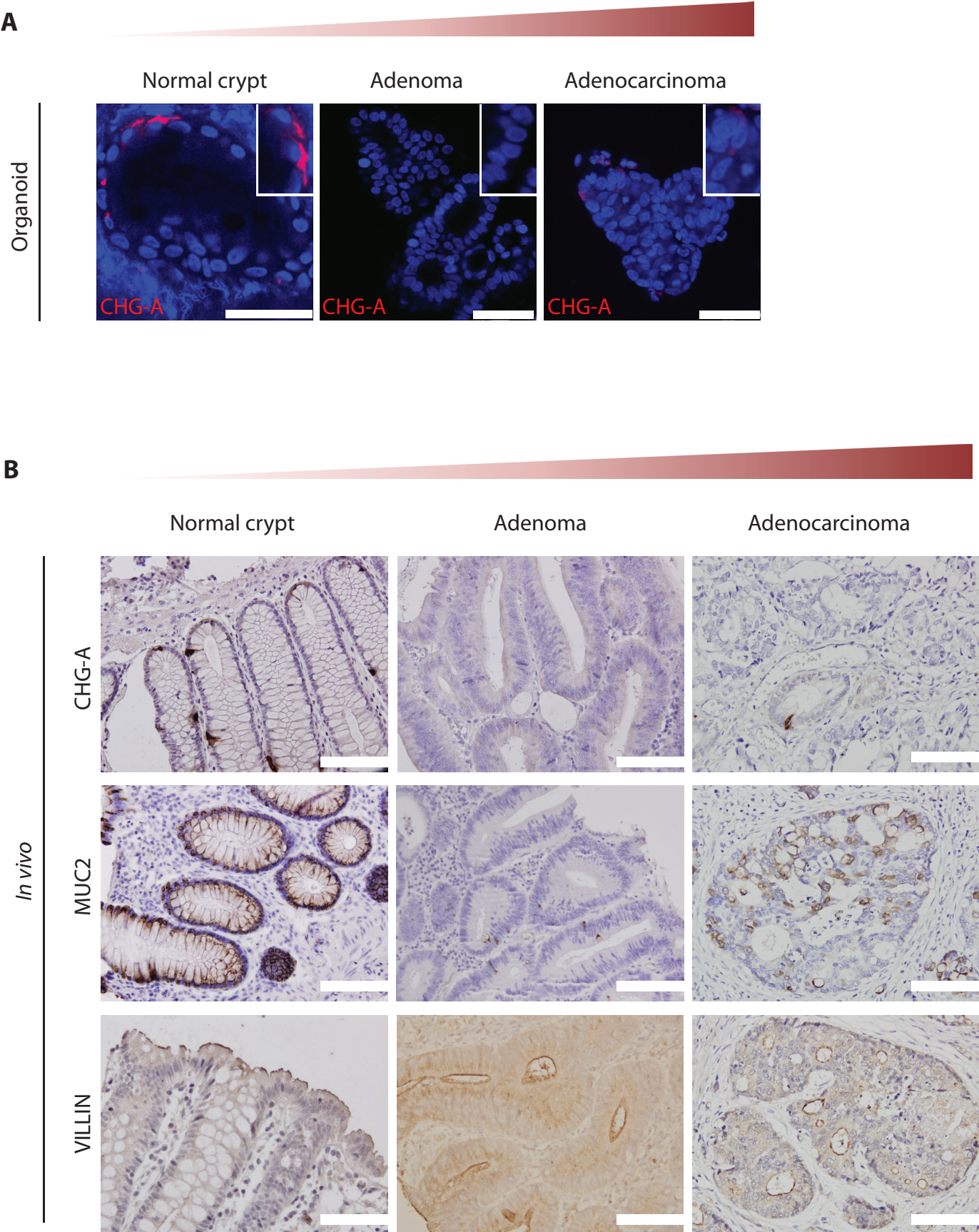

Supplement: Additional file 2: Figure S2 — Induction of goblet cells in mouse organoid cultures upon DBZ treatment in normal mouse epithelial cells and adenoma organoid cultures. [file 1476-4598-12-126-S2.pdf]
