# Supplementary material for: Regulation of stem cell self-renewal and differentiation by Wnt and Notch are conserved throughout the adenoma-carcinoma sequence in the colon
Source: Mol Cancer. 2013 Oct 21;12:126. doi: 10.1186/1476-4598-12-126 (PMC4016508; doi:10.1186/1476-4598-12-126)

Supplementary figure 2.
Goblet cell lineage induction in mouse organoid culture upon DBZ treatment

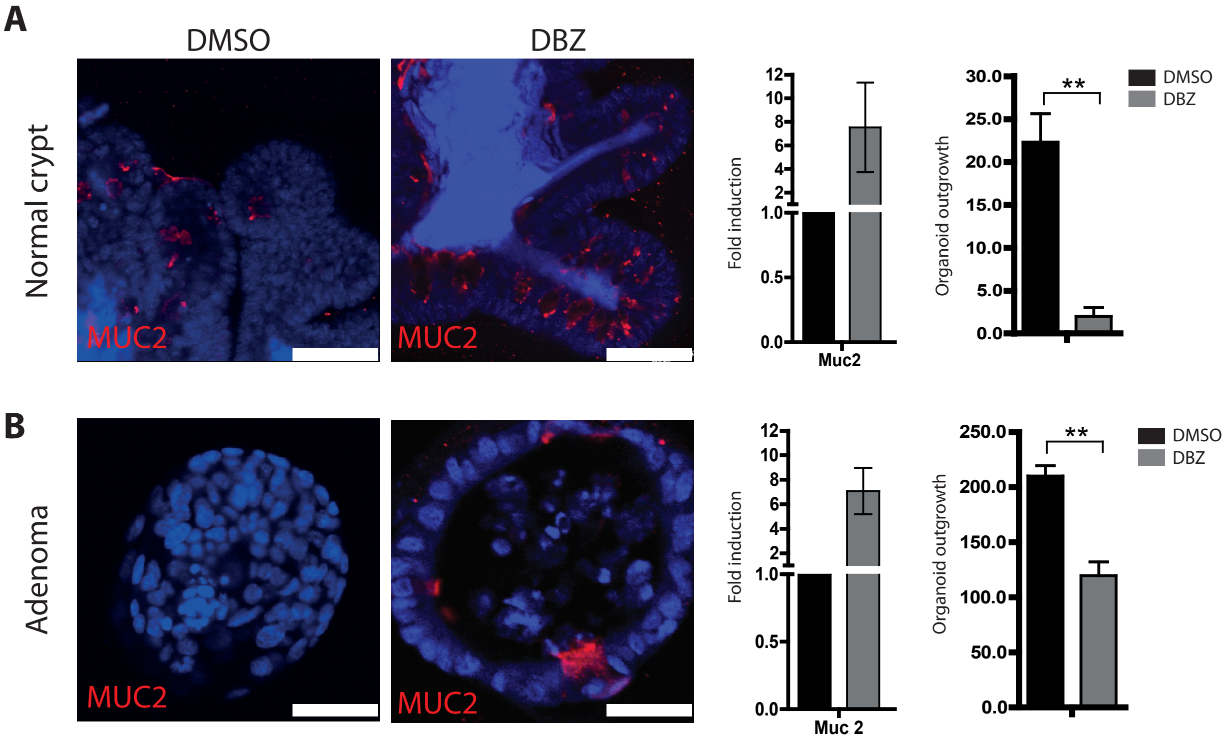

Supplement: Additional file 3: Figure S3 — Induction of goblet cells following DBZ treatment correlates with basal MUC2 expression. [file 1476-4598-12-126-S3.pdf]
